# Effect of Continuous Capacity Rising Performed by FeS/Fe_3_C/C Composite Electrodes for Lithium‐Ion Batteries

**DOI:** 10.1002/cssc.201903045

**Published:** 2020-02-06

**Authors:** Chengping Li, Angelina Sarapulova, Kristina Pfeifer, Sonia Dsoke

**Affiliations:** ^1^ Institute for Applied Materials Karlsruhe Institute of Technology Hermann-von-Helmholtz-Platz 1 76344 Eggenstein-Leopoldshafen Germany; ^2^ Helmholtz Institute Ulm for Electrochemical Energy Storage (HIU) Helmholtzstraße 11 89081 Ulm Germany

**Keywords:** electrocatalysis, batteries, carbides, iron, lithium

## Abstract

FeS‐based composites are sustainable conversion electrode materials for lithium‐ion batteries, combining features like low cost, environmental friendliness, and high capacities. However, they suffer from fast capacity decay and low electron conductivity. Herein, novel insights into a surprising phenomenon of this material are provided. A FeS/Fe_3_C/C nanocomposite synthesized by a facile hydrothermal method is compared with pure FeS. When applied as anode materials for lithium‐ion batteries, these two types of materials show different capacity evolution upon cycling. Surprisingly, the composite delivers a continuous increase in capacity instead of the expected capacity fading. This unique behavior is triggered by a catalyzing effect of Fe_3_C nanoparticles. The Fe_3_C phase is a beneficial byproduct of the synthesis and was not intentionally obtained. To further understand the effect of interconnected carbon balls on FeS‐based electrodes, complementary analytic techniques are used. Ex situ X‐ray radiation diffraction and ex situ scanning electron microscopy are employed to track phase fraction and morphology structure. In addition, the electrochemical kinetics and resistance are evaluated by cyclic voltammetry and electrochemical impedance spectroscopy. These results reveal that the interconnected carbon balls have a profound influence on the properties of FeS‐based electrodes resulting in an increased electrode conductivity, reduced particle size, and maintenance of the structure integrity.

## Graphical abstract

**Not fade away**: A FeS/Fe_3_C/C nanocomposite synthesized by a facile hydrothermal method is compared with pure FeS. When applied as anode materials for lithium‐ion batteries, these two types of materials show different capacity evolution upon cycling. Surprisingly, the composite delivers a continuous increase in capacity instead of the expected capacity fading. This unique behavior is triggered by a catalyzing effect of Fe_3_C nanoparticles.

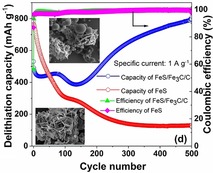

## 1 Introduction

The ever‐growing demand for portable electronic devices and large‐scale energy storage systems has promoted the great success of lithium‐ion batteries (LIBs) owing to their superior energy density, reliable stability, and long cycle lifetime. Further improvement of energy/power density and long cycling behavior of LIBs requires technological innovation of the electrode materials.^1^, ^2^, ^3^, ^4^ Promising negative electrode materials are based on a conversion mechanism.^5^ This mechanism results in significantly higher capacities of more than 1000 mAh g^−1[6]^ compared with, for example, graphite with a theoretical capacity of 372 mAh g^−1^.^7^ Iron sulfides have attracted much attention as electrodes for LIBs because of their high theoretical gravimetric capacities (609 mAh g^−1^ for FeS and 894 mAh g^−1^ for FeS_2_), natural abundance, and low cost.^8^, ^9^, ^10^ However, iron sulfide suffers from huge capacity decay owing to the sluggish electrode kinetics and drastic intrinsic volume expansion during the repeated lithiation and delithiation processes. It was reported that Li‐ion insertion into the FeS material results in a 200 % volume change, leading to electrode pulverization and thus to a drastic reduction of the electrical contact to the current collector.^11^ More deleteriously, insulating polysulfides Li_2_S_*x*_ (2<*x*<8) are formed during lithium‐ion insertion into iron sulfide materials.^12^, ^13^ Such polysulfides are partially soluble in organic electrolyte^14^ and can gradually form an insulating layer on the surface of the electrode, which seriously decreases the conductivity among active material particles and hinders further electrochemical reactions.^15^, ^16^

To solve the aforementioned problems, some useful strategies have been implemented: one is to improve the electrochemical performance by downsizing the particle size or fabricating nanostructures;^17^, ^18^ another strategy is to control the composition with carbon materials (such as carbon nanotubes,^19^ graphene,^20^ conductive polymers,^21^ and carbon fibers)^22^ to improve electron conductivity and structure stability. Two‐dimensional (2D) structures, such as flakes and sheets can effectively increase the contact between active materials and electrolyte, buffer the volumetric fluctuations, and decrease the diffusion length of lithium ions and electrons during the lithiation and delithiation processes.^17^, ^23^ For example, Xu et al.^16^ prepared carbon‐coated FeS nanosheets by a surfactant‐assisted solution‐based synthesis, which deliver excellent Li storage properties (615 mAh g^−1^ at a specific current of 100 mA g^−1^). The ultrathin FeS nanosheets can accommodate the volume expansion and shorten the diffusion paths. It is featured that the added carbon material can provide fast electron/ion transport and promote the formation of a stable solid electrolyte interphase (SEI) layer on the active material surface during the electrochemical processes.^9^, ^24^, ^25^ However, a detailed understanding of the kinetic phenomena and resistive contribution of FeS‐based materials during long‐term cycling and charge/discharge at high specific current (i.e., higher than 1 A g^−1^) is still not established. Moreover, the design of Fe_3_C‐containing composite materials was proposed as beneficial by different research groups. Su et al.^26^ presented a core–shell Fe@Fe_3_C/C composite and attributed the observed extra capacity beyond the carbon component to reversible redox reactions of some SEI components. These reactions were proposed to be catalyzed by Fe_3_C nanoshells. In another work by Zhang et al.,^27^ a similar positive contribution of Fe_3_C on Fe_3_O_4_@Fe_3_C core–shell nanoparticles was assigned to the stabilization of Fe_3_O_4_ particle integrity. What's more, Chan et al.^28^ prepared Fe/Fe_3_C/NPGC with high catalytic activity for enhanced bioelectricity generation. Both groups intentionally added the Fe_3_C component to their composite.

In this study, we have synthesized FeS nanosheets and FeS/Fe_3_C/C nanocomposites consisting of well‐dispersed FeS and Fe_3_C nanoparticles and interconnected carbon balls by a facile hydrothermal method and a subsequent sintering process. The Fe_3_C nanoparticles were formed as a byproduct but demonstrate a positive influence on the electrochemical performance. The FeS electrode continuously declines in capacity and exhibits a terrible capacity retention, whereas the capacity of the FeS/Fe_3_C/C electrode demonstrates a fluctuation prior to the 140th cycle and a continuous increase during further cycling. This is the first time that this kind of interesting behavior is observed. This manuscript focuses on a comprehensive and in‐depth investigation into the effect of the interconnected carbon balls–FeS and their property relationship. For this purpose, a series of electrochemical, physical, and morphological characterization techniques are employed to understand the influence of the interconnected carbon balls on FeS‐based electrodes.

## 2 Results and Discussion

### 2.1 Structural and morphological characterization

**Figure 1:**
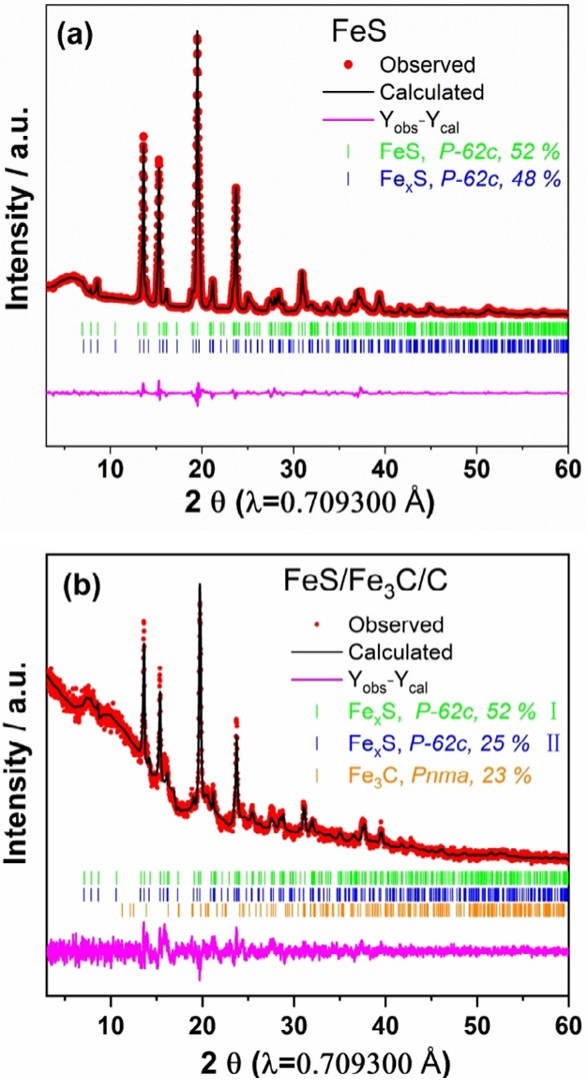
Rietveld refinement from X‐ray radiation diffraction data of (a) FeS and (b) FeS/Fe_3_C/C.

The phase fraction and crystal structure of FeS and FeS/Fe_3_C/C are evaluated by X‐ray diffraction (XRD) and analyzed by the Rietveld refinement by using the FullProf software package, as shown in Figure 1 a and b. The Rietveld refinement confirms that the XRD reflections of FeS are well indexed to the hexagonal FeS structure model with the same space group of *P* $\bar{6}$ 2*c*. The cell parameters are *a*=*b*=5.966 Å, *c*=11.738 Å for stoichiometric domains of FeS (52 %) and *a*=*b*=5.978 Å, *c*=11.535 Å for non‐stoichiometric domains of Fe_*x*_S (48 %). Moreover, FeS/Fe_3_C/C consists of two non‐stoichiometric phases of Fe_*x*_S and the Fe_3_C phase. For the Fe_*x*_S (I) phase (52 %), the cell parameters are *a*=*b*=5.959 Å, *c*=11.402 Å and for the Fe_*x*_S (II) phase (25 %) *a*=*b*=5.974 Å, *c*=11.486 Å. For the Fe_3_C phase (23 %), the cell parameters are *a*=5.007 Å, *b*=6.708 Å, *c*=4.297 Å.

**Figure 2:**
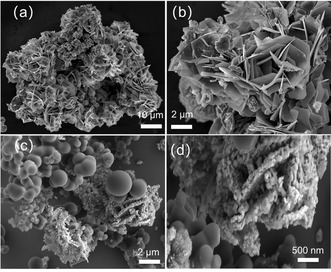
SEM images of (a, b) the FeS nanosheets and (c, d) FeS/Fe_3_C/C composites.

Scanning electron microscopy (SEM) images of FeS and FeS/Fe_3_C/C are given in Figure 2 a–d. The low‐ and high‐magnification SEM images of FeS (Figure 2 a,b) reveal that this material has the shape of nanosheets with an average size of 10 μm. These thin nanosheets stack together, forming three‐dimensional nanoflowers. The energy‐dispersive spectroscopy (EDS) elemental maps of FeS nanosheets are displayed in Figure S1 a–c (in the Supporting Information). The corresponding elemental mapping of Fe and S show a similar intensity distribution, implying that FeS is uniformly dispersed. The FeS/Fe_3_C/C composite is composed of FeS and Fe_3_C nanoparticles with a size of 50–60 nm and interconnected carbon balls of 1–2 μm (Figure 2 c,d). Herein, FeS nanoparticles are surrounded by interconnected carbon balls, which are expected to provide the paths for electron movement and effectively buffer the volume expansion upon repeated cycling. The corresponding elemental mapping of FeS/Fe_3_C/C (Figure S1 d–g in the Supporting Information) shows that the nanosized FeS nanoparticles are randomly scattered in the interconnected carbon ball matrix. To better understand the structure of FeS nanosheets and FeS/Fe_3_C/C composites, Raman spectroscopy of FeS and FeS/Fe_3_C/C were conducted and are shown in Figure S2 (in the Supporting Information). Both FeS nanosheets and FeS/Fe_3_C/C composites have two peaks located at 218 and 290 cm^−1^, which are attributed to the asymmetric and symmetric stretching modes of FeS.^29^ In the FeS/Fe_3_C/C composites, two distinct peaks are presented at 1315 and 1600 cm^−1^, which are related to the D band and G band of amorphous carbon with the intensity ration of *I*_D_/*I*_G_=1.0, implying that the interconnected carbon balls have a highly disordered carbon structure.^30^ The D band is linked to disordered carbon atoms and defects, whereas the G band is due to the relative motion of sp^2^ carbon atoms.^31^, ^32^ The carbon percentage in FeS/Fe_3_C/C is 55 %, which is calculated from the organic elemental analysis (OEA) measurements (see the Supporting Information, Table S1). These results reveal that the introduction of interconnected carbon balls in FeS drastically affects the phase fraction, the morphology, and the particle size.

### 2.2 Electrochemical performance and kinetic processes

**Figure 3:**
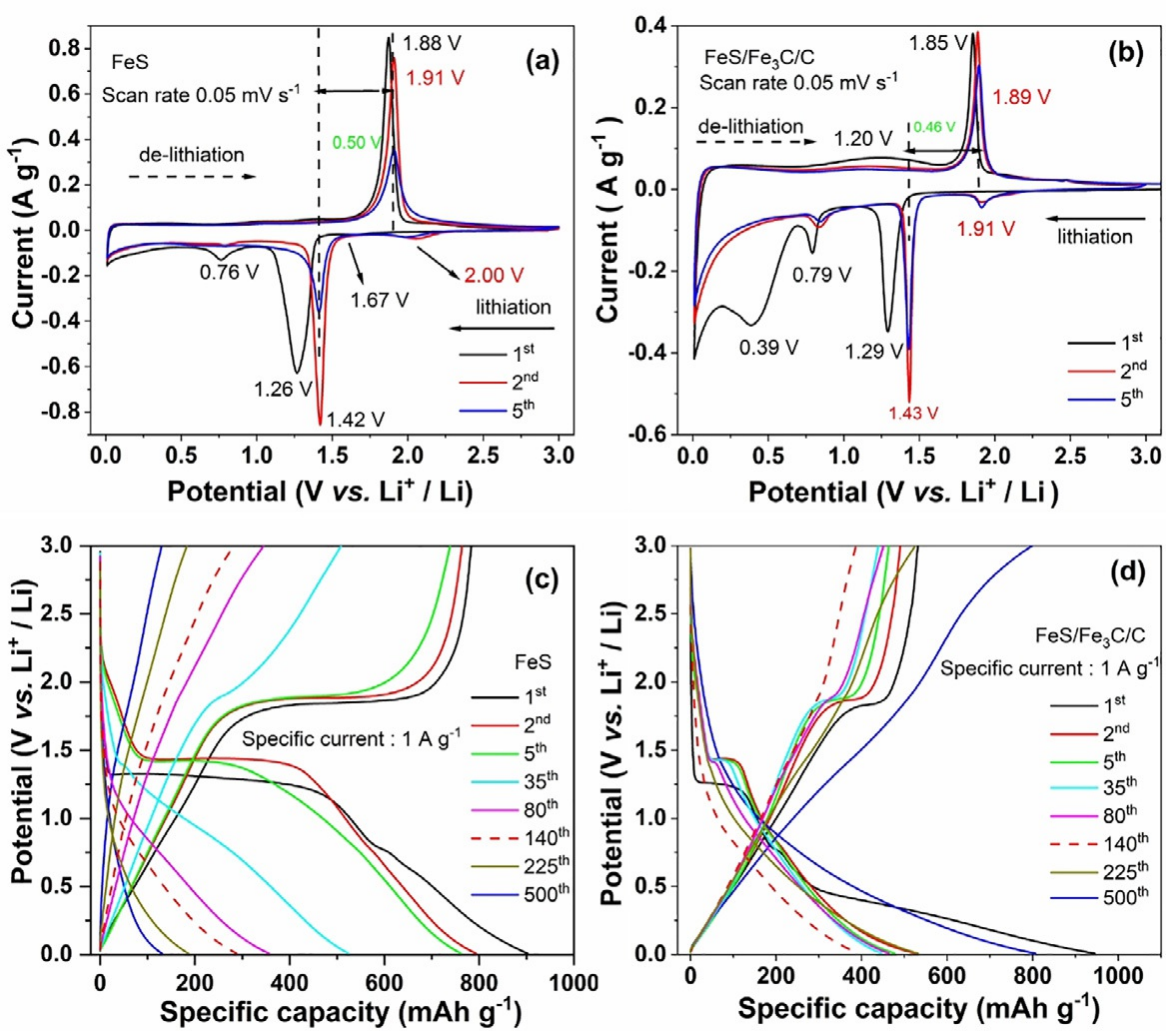
CV curves of (a) FeS and (b) FeS/Fe_3_C/C electrodes at a scan rate of 0.05 mV s^−1^. Galvanostatic lithiation/delithiation capacity profiles at different cycles for (c) FeS and (d) FeS/Fe_3_C/C electrodes at the specific current of 1 A g^−1^.

As huge disparities between the FeS nanosheets and FeS/Fe_3_C/C composites were observed by the previously described analytics, a decisively different electrochemical behavior is expected for the respective electrodes. To better understand the lithium‐storage mechanism taking place in the electrodes, cyclic voltammetry (CV) measurements were conducted at a scan rate of 0.05 mV s^−1^ in the voltage range from 0.01 to 3.0 V (vs. Li^+^/Li) for the FeS and FeS/Fe_3_C/C electrodes, respectively. Figure 3 a,b shows the CV profiles of the first five cycles.

The related equations from previous reports,^3^, ^17^, ^33^, ^34^, ^35^ representing state‐of‐the‐art lithium insertion in FeS and Fe_3_C are shown below:

(1) $$2\;FeS+2{\;Li}^{+}+2\;e^{-}\to {Li}_{2}FeS_{2}+Fe$$

(2) $$FeS+2{\;Li}^{+}+2\;e^{-}\to {Li}_{2}S+Fe$$

(3) $${Li}_{2}S+Fe-x\;Li-x{\;e}^{-}\to {Li}_{2-x}FeS_{2}$$

(4) $${Li}_{2-x}FeS_{2}+x{\;Li}^{+}+x\;e^{-}↔{Li}_{2}FeS_{2}$$

(5) $${Li}_{2}FeS_{2}+2\;Li\to 2{Li}_{2}S+Fe$$

(6) $${Fe}_{3}C+1/6\;Li↔{Li}_{\frac{1}{6}}C+3\;Fe$$

The lithium storage mechanism for Fe_3_C is based on a conversion mechanism; it is reported that only 1/6 Li per unit can insert into the Fe_3_C material (≈26 mAh g^−1^),^35^ and the phase fraction of Fe_3_C in FeS/Fe_3_C/C is 23 %. It is expected that the capacity contribution from Fe_3_C for the FeS/Fe_3_C/C electrode is negligible. Considering the CV of the FeS electrode, three reduction peaks appear at 1.7 V, 1.26 V, and 0.76 V, whereas only one oxidation peak appears at 1.88 V during the first scan. According to previous reports,^17^, ^33^, ^34^ the small peak at 1.7 V corresponds to the formation of the intermediate phase Li_2_FeS_2_ during the Li^+^ insertion into the FeS bulk [Eq. (1)]. The sharp peak at around 1.26 V is related to the conversion reaction belonging to the formation of metallic Fe nanocrystals and Li_2_S matrices [Eq. (2)].^16^ The broad peak at 0.76 V is assigned to the formation of a solid electrolyte interphase (SEI) on the electrode surface.^36^ In the first anodic process, the oxidation peak at 1.88 V corresponds to the oxidation of metallic Fe to form Li_2−*x*_FeS_2_ [Eq. (3)].^23^ In the subsequent cycles, the reduction peaks at 0.76 and 1.26 V shift to 0.79 and 1.42 V, respectively, and the oxidation peak at 1.88 V shifts to 1.91 V. These changes indicate that some irreversible reactions occur during the first electrochemical process. During the second to fifth cathodic scans, a new broad reduction peak appears at 2.0 V and can be related to the phase transformation from Li_2−*x*_FeS_2_ to Li_2_FeS_2_ [Eq. (4)].^15^ The sharp peak at 1.42 V corresponds to the lithiation process [Eq. (5)]. Correspondingly, the oxidation peak at 1.91 V in the second cycle accounts for the reversible delithiation process from Li_2_FeS_2_ to Li_2−*x*_FeS_2_ [Eq. (4)].^16^, ^17^ Upon the first five CV scans, the intensity of the redox peaks gradually decreases, indicating that the capacity decreases. This might result from unstable formation of the SEI layer and the sluggish reaction kinetics of pure FeS nanosheets.^3^, ^37^ The CV curves of the FeS/Fe_3_C/C electrode are similar to those of FeS except for the additional broad cathodic peak at 0.39 V, which is attributed to side reactions between the FeS/Fe_3_C/C material and the electrolyte and SEI formation.^2^ The conversion reaction between Fe_3_C and Li can be describe as Equation (6), with less capacity contribution. Moreover, the intensity of the reduction/oxidation peaks are much weaker owing to the interconnected carbon balls. Comparing the CV curves of FeS and FeS/Fe_3_C/C electrodes, the peak current densities and the peak potentials of the FeS/Fe_3_C/C electrode barely change after the first cycle. This points out that a better structural stability and good reversibility is accomplished for the FeS/Fe_3_C/C electrode. In addition, the polarization voltage of the FeS/Fe_3_C/C electrode is 0.46 V, which is lower than that of the FeS electrode (0.50 V). This demonstrates that the interconnected carbon ball morphology improves the conductivity of the FeS/Fe_3_C/C electrode, leading to a reduced electrode polarization.

Figure 3 c and d displays the lithiation/delithiation profiles of FeS and FeS/Fe_3_C/C electrodes at the first, second, fifth, 35th, 80th, 140th, 225th, and 500th cycle at a specific current of 1 A g^−1^. During the first lithiation of the FeS electrode, a long potential plateau at around 1.3 V and a short potential plateau at 0.8 V are observed, which correspond to the lithiation process forming Li_2_S, Fe, and the SEI layer formation, respectively. During the delithiation process, the long potential plateau at 1.8 V is related to the formation of Li_2−*x*_FeS_2_. All these potential plateaus are in agreement with the peaks observed in the CV curves. The FeS electrode delivers a first lithiation capacity of 900 mAh g^−1^ and a delithiation capacity of 782 mAh g^−1^ with a coulombic efficiency of 86.9 %. An irreversible capacity of 118 mAh g^−1^ at the first cycle results from the inevitable formation of the SEI film on the surface of the active material and electrolyte decomposition.^37^ In the second and fifth cycles, the reduction and oxidation plateaus shift to 1.4 and 1.9 V, respectively. The reason is an increased electrode polarization. In the second cycle, the FeS electrode delivers a delithiation capacity of 763 mAh g^−1^ whereas the coulombic efficiency increases to 96.6 %. After the fifth cycle, it still delivers a lithiation capacity of 760 mAh g^−1^ and a delithiation capacity of 738 mAh g^−1^ with the coulombic efficiency of 97.1 %. Examining the subsequent cycles of the FeS sample (the 35th, 80th, and 140th), a drop of the reduction plateau to lower potentials accompanied by a gradual decrease in delithiation capacity can be observed. This behavior is attributed to an enhancement of the electrode polarization during cycling. Furthermore, in the 225th and 500th cycles, no pronounced potential plateau is observed, implying structural changes or pulverization. After the 500th cycle, the voltage plateau disappears and the electrode delivers a very low delithiation capacity of 150 mAh g^−1^. This fact confirms that the structure of FeS nanosheets is destroyed. When comparing the FeS sample with the FeS/Fe_3_C/C one, the potential plateau of the FeS/Fe_3_C/C electrode is much shorter. During the first lithiation process, two plateaus are located at 1.3 and 0.8 V corresponding to the formation of Li_2_S, Fe, and SEI layers, respectively. A broad peak at 1.20 V corresponds to the oxidation of Fe^0^ and a very short potential plateau at 1.8 V related to Li_2−*x*_FeS_2_ formation is observed in the first delithiation process. In the first cycle, the FeS/Fe_3_C/C electrode shows lithiation and delithiation capacities of 946 and 530 mAh g^−1^, respectively, and a coulombic efficiency of 56 %. The huge irreversible capacity of 416 mAh g^−1^ is attributed to the SEI layer formation, electrolyte decomposition, and side reactions. In the subsequently cycles, the reduction plateau shifts from 1.3 to 1.4 V and the long slope disappears, implying that an irreversible reaction occurred. In the second cycle, it delivers a delithiation capacity of 490 mAh g^−1^ and the coulombic efficiency increases to 93 % whereas the fifth cycle shows a lithiation capacity of 476 mAh g^−1^ and a delithiation capacity of 463 mAh g^−1^ with a coulombic efficiency of 97 %. It becomes apparent that the length of the potential plateau decreases upon cycling (the 5th, 35th, 80th, and 140th cycles). At the 140th cycle, the potential plateau disappears and the electrode shows the lowest capacity of 395 mAh g^−1^ at this state. Interestingly, a capacity increase of the FeS/Fe_3_C/C electrode is observed upon further cycling. It is important to note that the lithiation/delithiation profiles after the 140th cycle with no clear plateau are strongly different from that of the first five cycles. Moreover, the specific capacity increases to 800 mAh g^−1^. According to the conversion mechanism for lithium storage, Fe_3_C can insert only 1/6 Li per unit (26 mAh g^−1^), which is negligible compared with the high capacity of 800 mAh g^−1^.^35^ This novel phenomenon has been observed for the first time and will be discussed hereafter in detail.

**Figure 4:**
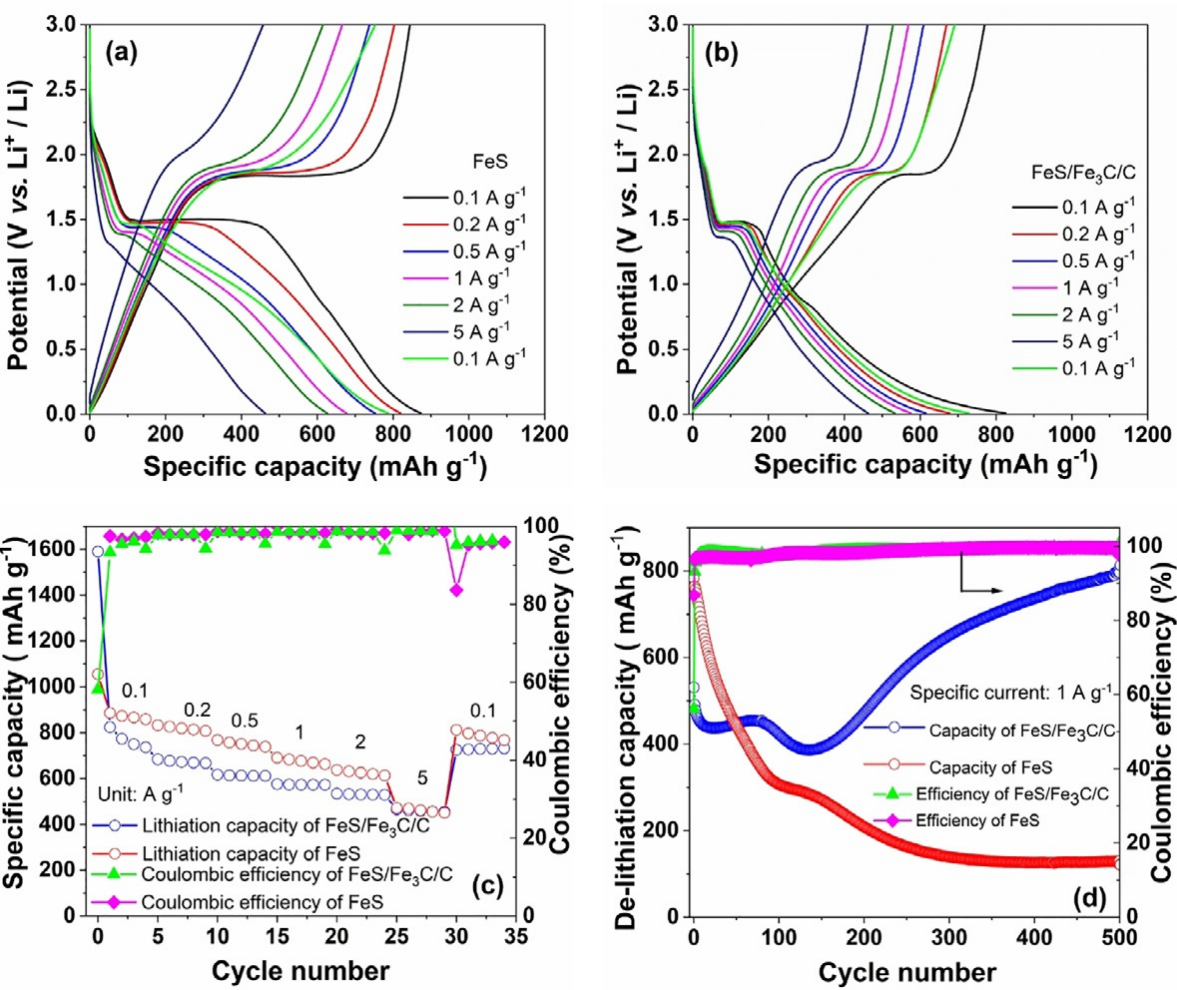
Lithiation and delithiation capacity profiles of (a) FeS and (b) FeS/Fe_3_C/C electrodes at different specific currents. (c) Rate performance of FeS and FeS/Fe_3_C/C electrodes. (d) Long‐term cycling and coulombic efficiency of FeS and FeS/Fe_3_C/C electrodes at specific current of 1 A g^−1^.

To evaluate the effect of interconnected carbon balls on FeS‐based electrodes, rate performances were applied at different specific currents in the voltage range from 0.01 to 3.0 V (vs. Li^+^/Li) for the FeS and FeS/Fe_3_C/C electrodes and are shown in Figure 4 a and b, respectively. On the one hand, the FeS electrode delivers 874, 819, 748, 674, 624, and 460 mAh g^−1^ at specific currents of 0.1, 0.2, 0.5, 1, 2, and 5 A g^−1^, respectively. When the specific current is set back to 0.1 A g^−1^, the specific capacity reaches 788 mAh g^−1^. On the other hand, the FeS/Fe_3_C/C electrode delivers 815, 676, 610, 572, 532, and 457 mAh g^−1^ at specific currents of 0.1, 0.2, 0.5, 1, 2, and 5 A g^−1^, respectively. As the specific current returns to 0.1 A g^−1^, the specific capacity returns to 724 mAh g^−1^. Figure 4 c directly compares the capacity values of FeS and FeS/Fe_3_C/C electrodes at the various specific currents. Between 0.1 and 2 A g^−1^, the FeS electrode displays higher capacities with respect to the FeS/Fe_3_C/C electrode. However, at the highest specific current (5 A g^−1^), both electrodes deliver the same specific capacity of 460 mAh g^−1^. Furthermore, the long‐term cycling performance of FeS and FeS/Fe_3_C/C electrodes were investigated at specific current of 1 A g^−1^ for the potential range 0.01–3.0 V (vs. Li^+^/Li; Figure 4 d). Both electrodes display a different behavior upon cycling. Despite the FeS electrode initially showing a much higher capacity compared with the FeS/Fe_3_C/C electrode, its capacity rapidly fades to 130 mAh g^−1^. Apart from that, the FeS/Fe_3_C/C electrode shows a capacity fluctuation during the first 140 cycles. After that, the capacity increases to 800 mAh g^−1^ after 500 cycles. A possible reason for such extra capacity could be the catalytic activation of Fe_3_C. It is reported that Fe_3_C plays the role of catalyst to decrease the concentration of SEI components and boost the reversible formation/decomposition of the SEI layer upon cycling.^26^, ^35^ In addition, the FeS/Fe_3_C/C particles shrink owing to pulverization, leading to higher electrochemical efficiency. Additionally, smaller particles can lead to higher capacities owing to less inactive material parts. Most plausible, the abovementioned trends result from the pseudo‐capacitive behavior of the material and therefore improve the electrochemical kinetics.

**Figure 5:**
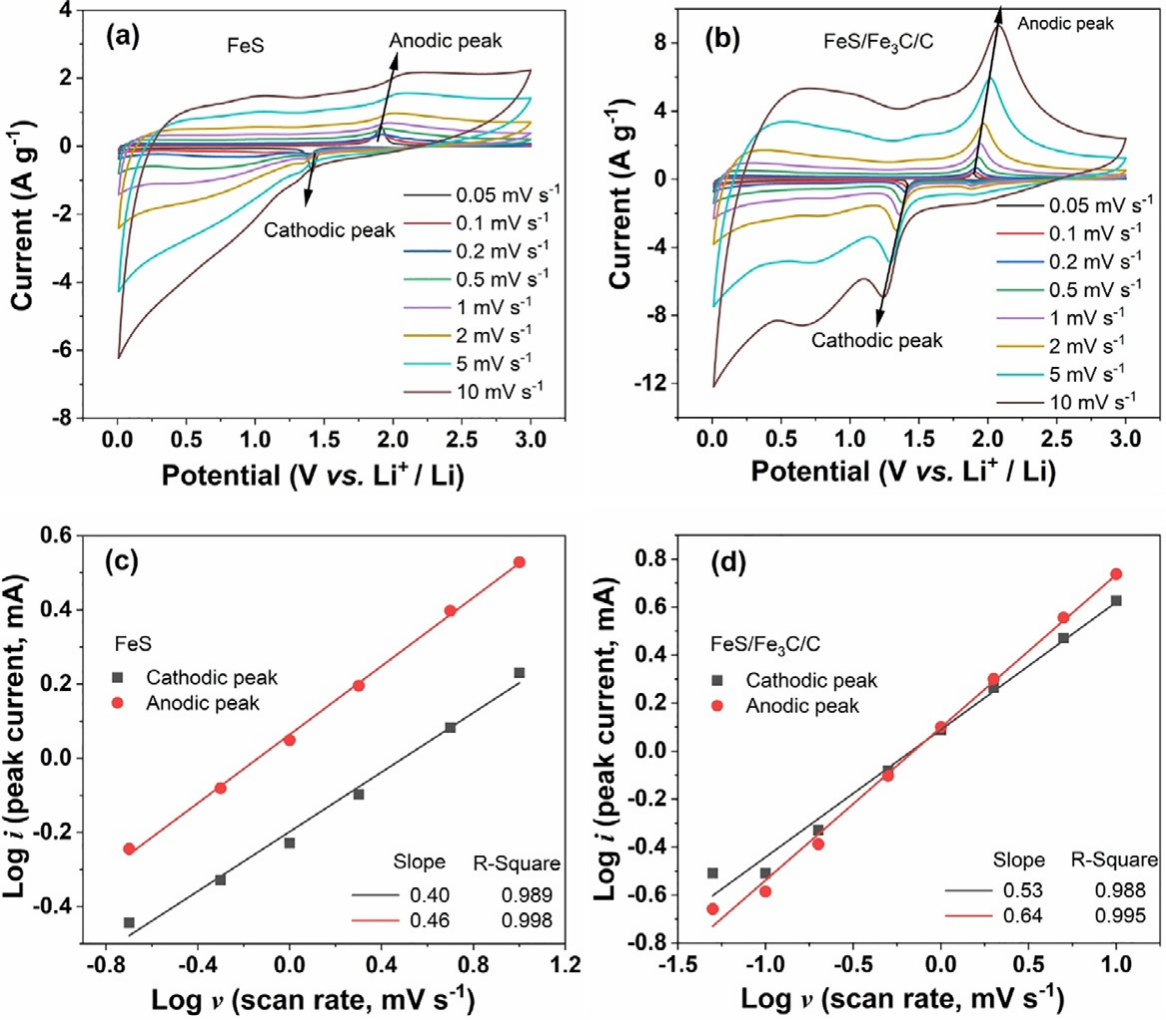
CV profiles at various scan rates ranging from 0.05 to 10 mV s^−1^ for (a) FeS and (b) FeS/Fe_3_C/C electrodes. The linear relationship between the log (*i*) and log (*v*) at cathodic and anodic peaks for (c) FeS and (d) FeS/Fe_3_C/C electrodes.

To prove the transformation of diffusion‐controlled behavior to a pseudo‐capacitive energy storage process after 140 cycles, CVs measurement were conducted at various scan rates. This experiment should allow insights into the storage mechanism during the initial cycling. Figure 5 a and b shows the CV profiles of the FeS and FeS/Fe_3_C/C electrodes at scan rates between 0.05 and 10 mV s^−1^. As expected, the peak current increases with the increase of sweep rate. The current (*i*) is related to the scan rate (*v*) through the relation: $i=av^{b}$ . Generally, *b*=0.5 implies a diffusion‐controlled process, whereas *b*=1 represents a capacitive process.^38^ The cathodic and anodic peaks in Figure 5 a and b were chosen to calculate the *b* value by using the equation log (*i*)=*b* log (*v*)+log (*a*).^39^ Based on the value of *b*, we can distinguish if the lithiation and delithiation are diffusion or surface controlled. Figure 5 c,d presents the linear relationship between the log (*i*) and log (*v*) at cathodic and anodic peaks for FeS and FeS/Fe_3_C/C electrodes, respectively. After linear fitting, the calculated *b* value of the cathodic and anodic peaks for the FeS electrode are 0.40 and 0.46, respectively; whereas those for the FeS/Fe_3_C/C electrode are 0.53 and 0.64, respectively. As expected, this analysis confirms that the ion‐diffusion behavior controls the electrochemical process in both FeS and FeS/Fe_3_C/C electrodes for the initial cycles. The diffusion‐controlled mechanism can explain the initial capacity fading. Enhanced stress is applied to the FeS active material by diffusion compared with a surface‐controlled process. Therefore, an initial pulverization and phase amorphization of both samples is expected, leading to a contact loss between the particles, which results in an inferior percolation and increased resistance. We expect that the interconnected carbon ball matrix of the FeS/Fe_3_C/C sample buffers the pulverization and allows a stable change to a pseudo‐capacitive mechanism during cycling. In this case, the interconnected carbon balls undertake the role of a conductive matrix with pulverized FeS nanoparticles distributed on it. These particles exhibit pseudo‐capacitive behavior, which is indicated by the loss of the plateaus and the simultaneous capacity increase. To prove this interpretation, further cycling experiments and ex situ measurements were conducted and are presented in the following section.

### 2.3 Phase fraction, morphology, and electrochemical performance of the electrode after cycling

**Figure 6:**
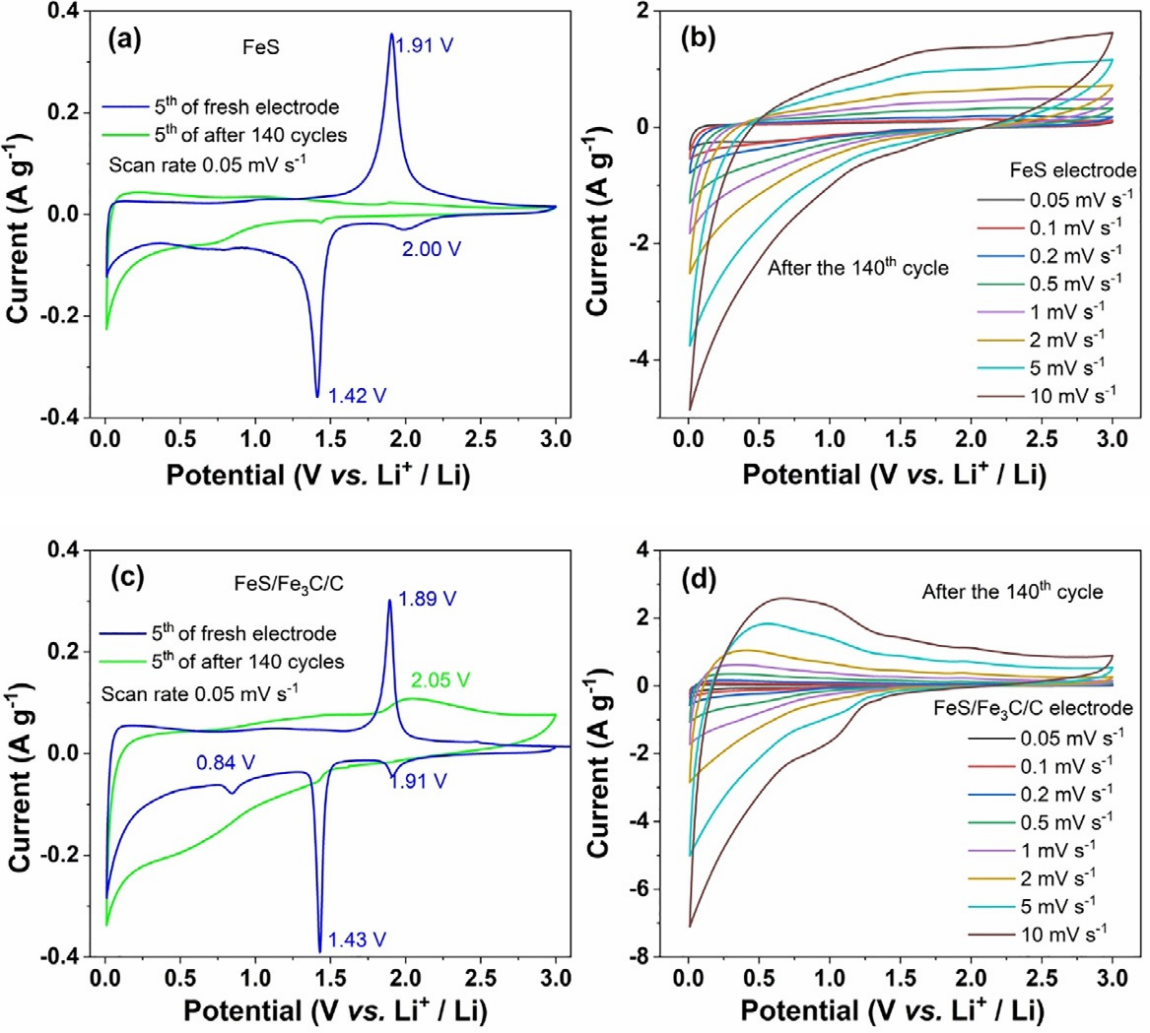
The fifth CV curves of the fresh electrode and after the 140th cycle for (a) FeS and (c) FeS/Fe_3_C/C electrodes. CV profiles at different sweep rates between 0.05 and 10 mV s^−1^ for (b) FeS and (d) FeS/Fe_3_C/C electrodes.

The above results highlight that the lithium storage mechanism strongly changes during cycling. To understand this mechanism, the FeS and FeS/Fe_3_C/C electrodes were analyzed after the 140th galvanostatic cycle by complementary techniques such as CV, ex situ XRD, and SEM. To understand the differences between fresh and cycled electrodes, CV measurements were performed after different cycle numbers and are shown in Figure 6 a and c. It can be observed that after the 140th cycle at 1 A g^−1^ the redox peaks become weaker for both the FeS (Figure 6 a) and the FeS/Fe_3_C/C electrode (Figure 6 c). This demonstrates an unstable cycling feature resulting from irreversible phase transitions and pulverization. It is noted that in the FeS/Fe_3_C/C electrode, the oxidation peak becomes broader and shifts (from 1.89 to 2.05 V) after 140 cycles, which indicates that the FeS/Fe_3_C/C electrode has higher resistance upon cycling. This result was analyzed in depth by electrochemical impedance spectroscopy (EIS) measurements and will be shown later (Figure 10 b,d). The oxidation/reduction peaks nearly disappear for the FeS electrode, implying that the faradaic reaction does not exist anymore. More interestingly, the cathodic peak (located at 0.84 V) of the FeS/Fe_3_C/C electrode appears at the fifth CV cycle of the fresh electrode owing to the formation of the SEI. However, there is a very broad cathodic peak in the fifth CV recorded after 140 cycles, which is related to some irreversible reactions. Figure 6 b and d present the CVs of FeS and FeS/Fe_3_C/C electrodes at different sweep rates after the 140th cycles. Cycled electrodes show no clear peaks compared with that of fresh ones (Figure 5 a,b). This confirms that the amorphous phase exists in the cycled electrode.

**Figure 7:**
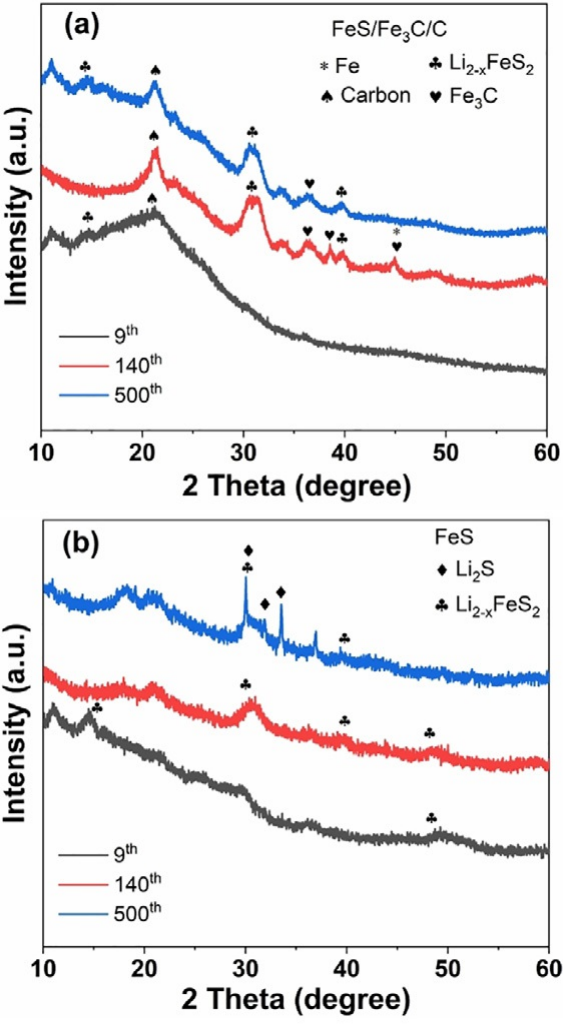
Ex situ XRD reflection patterns of (a) FeS/Fe_3_C/C and (b) FeS electrodes at the ninth, 140th, and 500th cycles (*λ*=1.5406 Å).

To further investigate the phase fraction and phase transition during the repeated lithiation/delithiation processes, ex situ XRD of the FeS/Fe_3_C/C and FeS electrodes at the 9th, 140th, and 500th cycles was performed and are shown in Figure 7 a,b. The FeS and FeS/Fe_3_C/C show sharp and clear XRD reflection patterns (Figure 1 a,b), whereas XRD reflection patterns of the cycled FeS/Fe_3_C/C electrode show broad peaks, corresponding to Li_2_S and Li_2−*x*_FeS_2_. The reason behind this is that during repeated lithiation and delithiation processes, the material becomes amorphous with small crystallite size. In contrast, the XRD reflection of the FeS electrode becomes sharper (such as 30°, 32°, and 37°), indicating that the particle size increases upon cycling.

**Figure 8:**
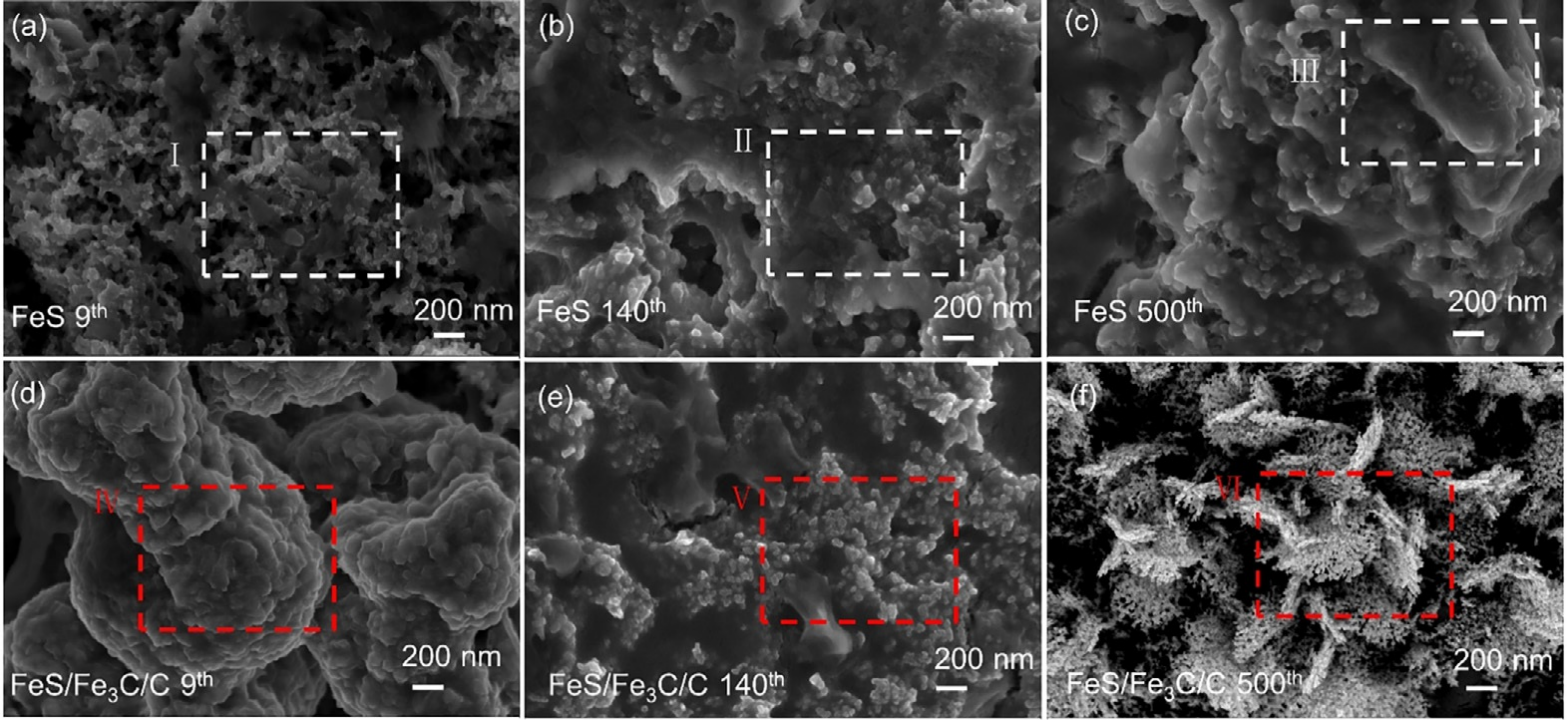
Morphological and structural changes of the FeS electrode at the (a) ninth, (b) 140th, and (c) 500th cycles. The corresponding ex situ SEM of the FeS/Fe_3_C/C electrode at the (d) ninth, (e) 140th, and (f) 500th cycles.

To further demonstrate the reason behind the electrochemical performance difference between FeS and FeS/Fe_3_C/C electrodes, the morphological changes of the cycled FeS and FeS/Fe_3_C/C electrodes are investigated by ex situ SEM and are shown in Figure 8. Figure 8 a–c shows the ex situ SEM of the FeS electrode at the ninth, 140th, and 500th cycles. Compared with the fresh FeS pristine material (Figure 2 a,b), the morphology of the cycled FeS particle undergoes an irreversible change. Comparing the ex situ SEM images of the FeS electrode at different cycles, it is interesting that some small clusters of FeS particles tend to agglomerate and form a large bulk, especially in the regions I, II, and III. The ex situ SEM image of the ninth cycle is composed of nanoparticles and many holes (region I, Figure 8 a), whereas the FeS particles crowd together and the holes disappear after the 140th cycle (region II, Figure 8 b). Finally, the particles further agglomerate, forming a large bulk at the 500th cycle (region III, Figure 8 c). The nanoparticle agglomeration is not beneficial for the reaction between the active material and electrolyte during cycling; this can explain the specific capacity decrease of the FeS electrode upon cycling (Figure 4 d). Moreover, one can see that the ex situ SEM image of the FeS/Fe_3_C/C particle at the ninth (Figure 8 d) cycle shows the large clusters agglomerate, which are wrapped with SEI films similar to the FeS electrode. The particles transform into smaller ones (Figure 8 e,f, at the 140th and 500th cycles) and tend to interconnect with each other upon cycling. These smaller particles are equally distributed with the interconnected carbon balls, which can enlarge the contact between the active material and electrolyte, thus resulting in the high efficiency of the electrochemical reaction, which is the most probable reason for the capacity increase upon cycling.

### 2.4 Electrochemical impedance spectroscopy evolution

**Figure 9:**
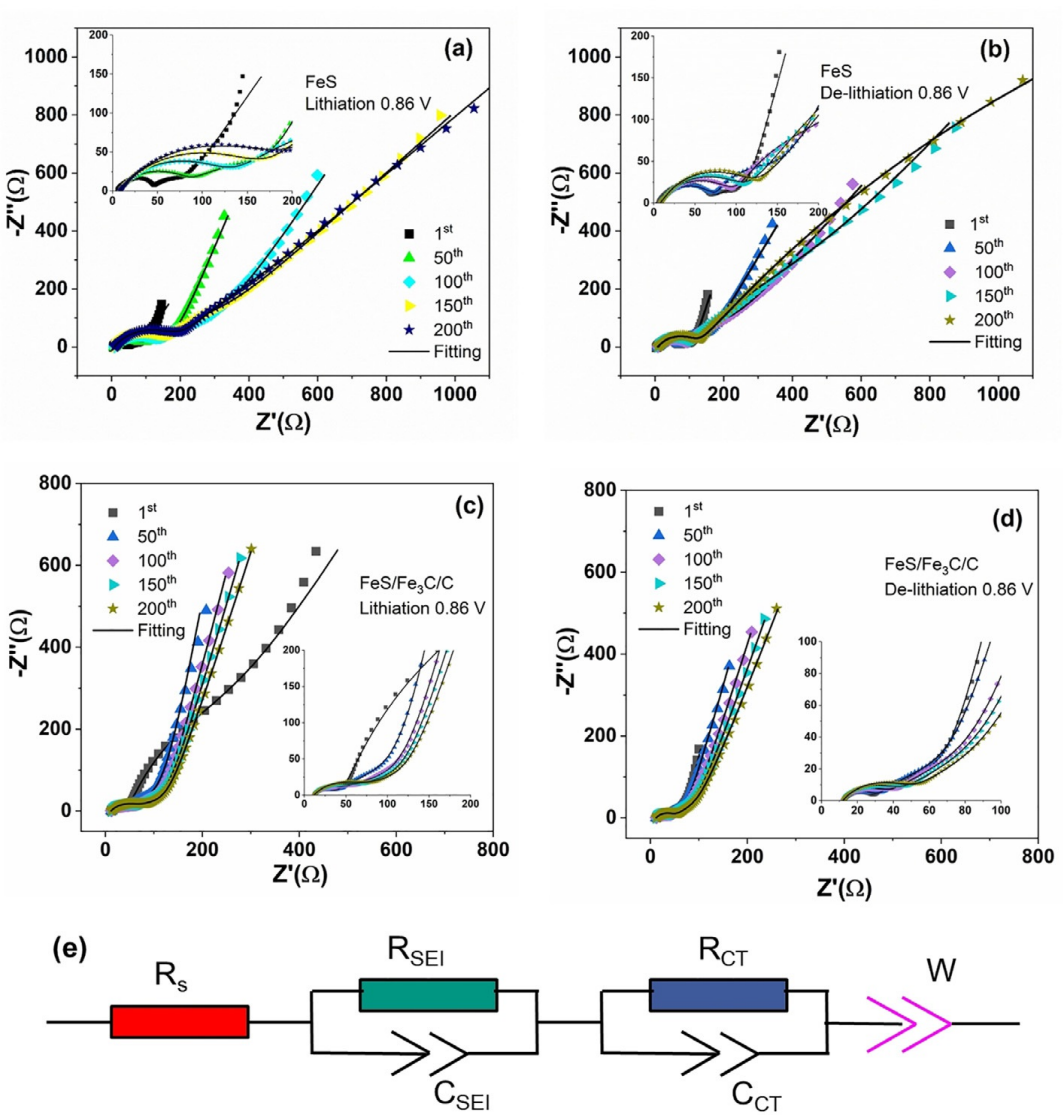
Nyquist plots of the FeS electrode at different cycles in (a) lithiation and (b) delithiation conditions (0.86 V). Nyquist plots of the FeS/Fe_3_C/C electrode at selected cycles in (c) lithiation and (d) delithiation states (0.86 V). The inset shows the zoom of Nyquist plots in the high‐frequency region. (e) The equivalent circuit used to fit the EIS data.

EIS was performed to examine the kinetics of Li^+^ insertion/de‐insertion processes upon cycling. Figure 9 a and b shows the Nyquist plots of the FeS electrode at different cycles (1st, 50th, 100th, 150th, and 200th) at the bias potential of 0.86 V (vs. Li^+^/Li) during lithiation and delithiation processes, respectively; correspondingly, the Nyquist plots of the FeS/Fe_3_C/C electrode are shown in Figure 9 c,d. The inset pictures in Figure 9 display the zoom in on the high‐frequency area. It is found that the EIS dispersions present common characteristics: (1) a small semicircle in the high‐frequency region, corresponding to the passivation layer; (2) a partially overlapped semicircle in the intermediate‐frequency region, corresponding to the charge‐transfer process and charge accumulation at the electrical double layer; (3) a sloping line in the low‐frequency region, which corresponds to the solid diffusion of lithium into the nanoparticle.^40^, ^41^ The Nyquit plots of FeS and FeS/Fe_3_C/C electrodes were fitted by using an equivalent circuit described as *R*_el_(*R*_SEI_ *C*_SEI_) (*R*_CT_ *C*_CT_)*W* in Boukamp's notation^42^ and shown in Figure 9 e. *R*_el_ represents the electrolyte resistance (including separator and internal connections), *R*_SEI_ and *C*_SEI_ are assigned to SEI resistance and capacitance, *R*_CT_ and *C*_CT_ are related to the charge‐transfer resistance and double layer capacitance, and *W* (alfa=0.5) is attributed to the Warburg impedance. It is worth noting that the overall impedance of the FeS and FeS/Fe_3_C/C electrodes show a resistance increase in both lithiation and delithiation conditions upon cycling. Comparing the Nyquist plots of the FeS and FeS/Fe_3_C/C electrodes at some selected cycles (i.e., the 1st, 100th, and 200th) in lithiation and delithiation conditions (Figure S3 in the Supporting Information), it is observed that the diameter of the semicircle for the FeS/Fe_3_C/C electrode is smaller than that for the FeS electrode. This demonstrates that the FeS/Fe_3_C/C electrode has rapid electrochemical reaction kinetics, which benefits from the addition of the interconnected carbon balls. Moreover, the slope in the low‐frequency region for the FeS/Fe_3_C/C electrode is larger than that for the FeS electrode, which implies faster Li^+^ mobility in the FeS/Fe_3_C/C electrode.^43^, ^44^

**Figure 10:**
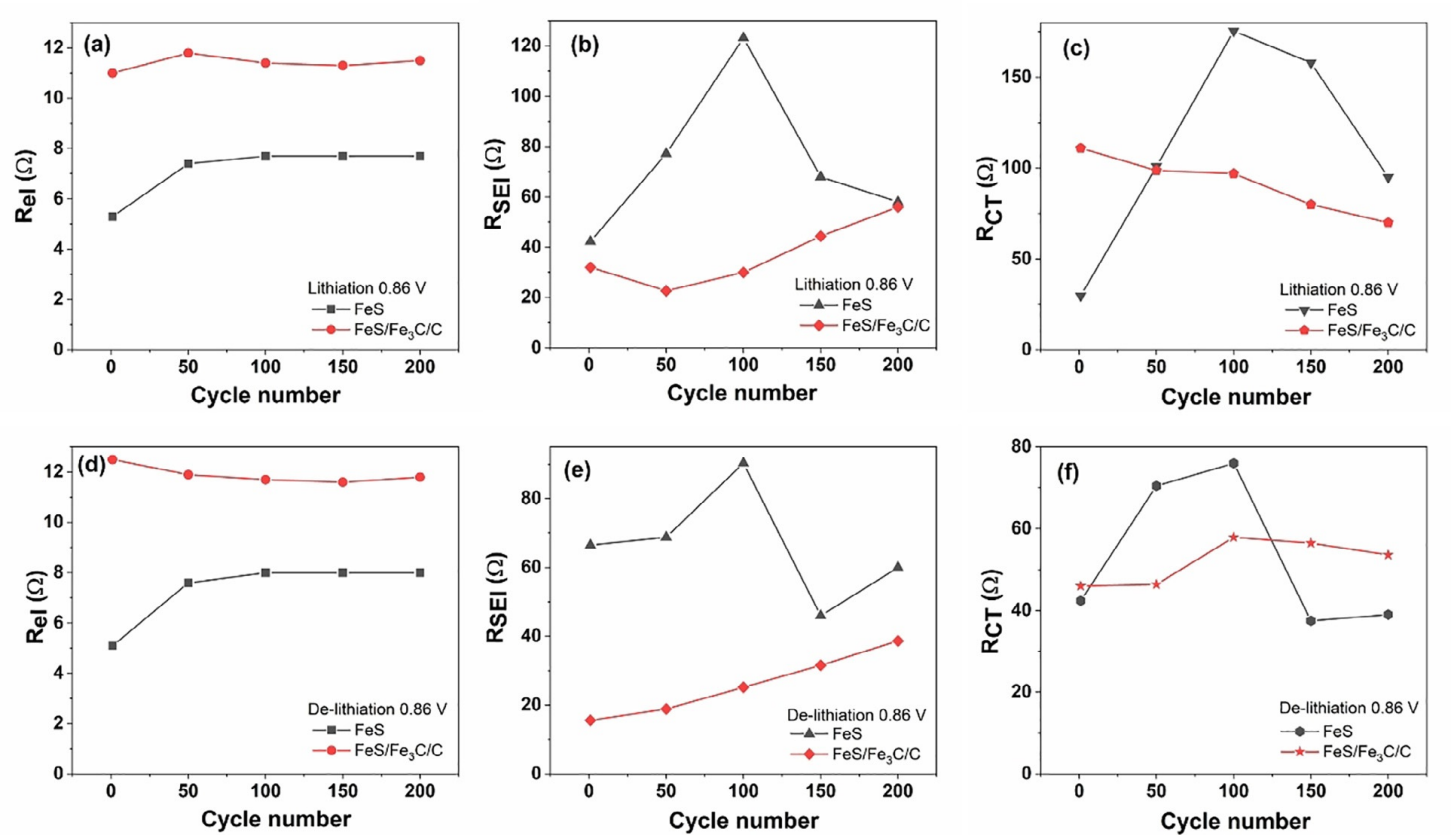
Calculated resistance values for FeS and FeS/Fe_3_C/C electrodes in lithiation conditions: (a) *R*_el_, (b) *R*_SEI_, (c) *R*_CT_; in delithiation conditions: (d) *R*_el_, (e) *R*_SEI_, and (f) *R*_CT_.

Figure 10 displays *R* values as calculated with Relaxis 3 software in lithiation and delithiation conditions. The electrolyte resistance *R*_el_ for the FeS/Fe_3_C/C electrode (11 Ω) is almost unchanged upon cycling, whereas the *R*_el_ for the FeS electrode first increases until the 100th cycle, then it remains quite stable at 8 Ω (Figure 10 a,d). The small difference of the *R*_el_ value between the FeS/Fe_3_C/C and FeS electrode is probably due to connections inside the cell. The calculated value of *R*_SEI_ is shown in Figure 10 b,e. For the FeS/Fe_3_C/C electrode, *R*_SEI_ slightly increases in both lithiation and delithiation conditions upon cycling. Furthermore, the *R*_SEI_ in lithiation conditions is higher than that in the delithiation state, indicating the dynamic nature of the SEI layer, which grows in the lithiation process and partially decomposes in the delithiation process.^45^, ^46^ The *R*_SEI_ of the FeS electrode drastically increases during the first 100 cycles, then it decreases but remains still higher than that of the FeS/Fe_3_C/C electrode. It is demonstrated that Fe_3_C exhibits great activity in promoting the partially reversible formation/decomposition of the SEI layer.^27^ *R*_CT_ of the FeS/Fe_3_C/C electrode slightly decreases in lithiation conditions upon cycling, and the *R*_CT_ value remains almost stable in delithiation conditions. The rapid charge‐transfer kinetics of FeS/Fe_3_C/C may benefit from a partial nanoparticle reaggregation, which mostly occurs during the initial cycles (Figure 8 c) whereas the morphology appears stabilized upon further cycling.^47^ In contrast, *R*_CT_ of the FeS electrode sharply increases until the 100th cycle, corresponding to the terrible capacity decay (Figure 4 d); in subsequent cycles, *R*_CT_ of the FeS electrode continuously declines but is still higher than that of the FeS/Fe_3_C/C electrode in lithiation conditions. In summary, the interconnected carbon ball morphology can improve Li^+^/electron mobility and form a better protective SEI layer, thus promoting the redox reaction.^48^

## Conclusion

FeS nanosheets and FeS/Fe_3_C/C nanocomposites consisting of well‐dispersed FeS and Fe_3_C nanoparticles and interconnected carbon balls were synthesized by a facile hydrothermal method and a subsequent sintering process. The interconnected carbon balls are found to have a significant impact on the electrochemical performance of the FeS‐based electrodes. We highlight the catalytic activity of Fe_3_C, which was formed as a beneficial byproduct during the conducted synthesis. Owing to the unique formulation of the composite, the electrochemical cycling performance is significantly enhanced. This is accompanied by a continuous increase in capacity. To understand the different lithium storage mechanisms and evaluate the effect of interconnected carbon balls on FeS‐based electrodes, some techniques such as CV, ex situ XRD, and SEM were applied. We discovered that the introduction of interconnected carbon balls in FeS drastically affects the phase fraction, morphology, and particle size. More importantly, the interconnected carbon balls have a profound influence on the kinetic process and crystal structure during cycling. Furthermore, such carbon balls change the diffusion‐controlled behavior to a pseudo‐capacitive energy storage process. Indeed, the interconnected carbon balls improve the electron conductivity, reduce the crystal size, and maintain the structural integrity. Especially for long cycling procedures, the well‐distributed FeS nanoparticles with small average diameters provide sufficient electrode–electrolyte contact areas for high lithium ion flux across the interface. A reduction of the lithium ion diffusion length during cycling significantly promotes the electrochemical processes, especially at high specific current.

## Experimental Section

### Synthesis of FeS nanosheets

Iron chloride hexahydrate (1.35 g, FeCl_3_ **⋅**6 H_2_O, Alfa Aesar, 99 %), polyacrylamide (0.2 g, (C_3_H_5_NO)_*n*_, Sigma–Aldrich), and thiourea (0.9 g, CH_4_N_2_S, Sigma–Aldrich, 99 %) were dissolved in deionized (DI) water (80 mL). The mixture was kept under continuous stirring for 60 min at room temperature. Then, the solution was transferred into a 100 mL Teflon‐lined autoclave and heated at 180 °C for 12 h. After washing with DI water several times, the collected black power was finally annealed in Ar/H_2_ (Ar/H_2_=95:5) at 600 °C for 5 h with a heating rate of 10 °C min^−1^.

### Synthesis of FeS/Fe_3_C/C composite material

d‐Glucose (630 mg, C_6_H_12_O_6_, Sigma–Aldrich, 99.5 %) was dissolved in DI water (40 mL). The above harvested FeS nanosheets (75 mg) were dispersed into the d‐glucose solution. Subsequently, the solution was transferred into a 50 mL Teflon‐lined autoclave and heated at 180 °C for 12 h. After cooling down to room temperature, the product was poured out and washed by using rinse–precipitation cycles with DI water. The product was dried at 80 °C. Finally, the harvested black powder was kept in Ar/H_2_ (Ar/H_2_=95:5) at 600 °C for 5 h with a heating rate of 10 °C min^−1^.

### Materials characterization

The crystallographic information and phase fraction of the FeS and FeS/Fe_3_C/C were obtained from a STOE STADI P COMBI diffractometer (Mo_Kα1_, *λ*=0.709300 Å) in Debye–Scherrer geometry. The morphology and composition of the sample were observed by using a thermal field‐emission scanning electron microscope (FESEM, Carl Zeiss SMT AG) equipped with energy‐dispersive spectroscopy (EDS, Quantax 400 SDD, Bruker). Raman spectra were collected with a LabRam Evolution HR in Via Raman spectrometer with a laser source (*λ*=532 nm, 10 mW, HORIBA Jobin Yvon). The carbon percentage in the composite was analyzed by using organic elemental analysis (OEA, Vario Micro Cube, Elementar).

### Electrochemical characterization

Electrochemical measurements were conducted by using three‐electrode Swagelok‐type half cells assembled in an argon‐filled glovebox (MBraun, O_2_ and H_2_O≤2 ppm). The working electrodes were prepared by mixing the active material, carbon black (super P Li, Timcal Ltd.), and polyvinylidene fluoride binder (PVDF) with a mass ratio of 7:2:1 in *N*‐methyl‐2‐pyrrolidone solvent (NMP) to form a homogeneous slurry. The slurry was stirred overnight at room temperature and then coated on a copper foil and dried at 80 °C. The circular working electrodes with a diameter of 12 mm were punched out and dried under vacuum at 120 °C for 24 h. The mass loading of active materials was 1.8 mg cm^−2^ for FeS and 0.7 mg cm^−2^ for FeS/Fe_3_C/C. The FeS/Fe_3_C/C electrodes are normalized to the entire mass of the composite (FeS/Fe_3_C/C) as active material. A lithium metal foil was used as the counter electrode and another as the reference electrode. The working and counter electrodes were separated by two 12 mm diameter vacuum‐dried glass‐fiber discs (Whatman GF/D). 1 m LiPF_6_ dissolved in ethylene carbonate/dimethyl carbonate (LP30, EC/DMC=1:1 in volume, BASF, Germany) was used as the electrolyte. Cyclic voltammetry (CV), galvanostatic cycling with potential limitation (GCPL), and electrochemical impedance spectroscopy (EIS) were conducted by using a multichannel potentiostat (VMP3, Bio‐Logic) in the voltage window range 0.01–3.0 V vs. Li^+^/Li. All electrochemical measurements were performed in a climate chamber (Binder) at 25 °C. CV was recorded at different scan rates in the range between 0.05 and 10 mV s^−1^. GCPL was conducted at different specific currents between 0.1–5 A g^−1^. Long‐term cycling was performed at 1 A g^−1^ for 500 cycles. Ex situ X‐ray radiation diffraction (XRD) and ex situ SEM measurements were performed on cycled electrodes (i.e., cycled at 1 A g^−1^, after the 9th, 140th, and 500th cycles) in the delithiation state. The cycled electrodes were disassembled and washed with dimethyl carbonate (DMC, Sigma–Aldrich, 99 %) in an argon‐filled glovebox. The ex situ XRD was measured with a STOE STADI P diffractometer (Cu_Kα1_, *λ*=1.5406 Å) in flat‐sample transmission mode. EIS experiments with a working electrode of 7 mm diameter were conducted at various selected potentials in the frequency range between 10 mHz and 500 kHz every 50 cycles. The cells were equilibrated at the desired potential for 3 h before recording the EIS data. The impedance spectra were analyzed by using Relaxis 3 software (rhd Instruments, Germany).

The EDS elemental maps of the pristine FeS material and FeS/Fe_3_C/C material, Raman, and OEA of the pristine FeS material and FeS/Fe_3_C/C material, the Nyquist plots of FeS and FeS/Fe_3_C/C electrodes at some selected cycles (1st, 100th, and 200th) in lithiation and de‐lithiation conditions are given in the Supporting Information.

## Conflict of interest

*The authors declare no conflict of interest*.

## Supporting information

As a service to our authors and readers, this journal provides supporting information supplied by the authors. Such materials are peer reviewed and may be re‐organized for online delivery, but are not copy‐edited or typeset. Technical support issues arising from supporting information (other than missing files) should be addressed to the authors.

Supplementary
