# Supplementary material for: Effect of Continuous Capacity Rising Performed by FeS/Fe3C/C Composite Electrodes for Lithium‐Ion Batteries
Source: ChemSusChem. 2020 Feb 6;13(5):986–95. doi: 10.1002/cssc.201903045 (PMC7079246; doi:10.1002/cssc.201903045)
Supplement: Supplementary file 1 — Supplementary [file CSSC-13-986-s001.pdf]

CHEMISTRY & SUSTAINABILITY
CHEMSUSCHEM
ENERGY & MATERIALS

Supporting Information

Effect of Continuous Capacity Rising Performed by $FeS/Fe_3C/C$ Composite Electrodes for Lithium-Ion Batteries

Chengping Li,[a] Angelina Sarapulova,[a] Kristina Pfeifer,[a] and Sonia Dsoke*[a, b]

cssc_201903045_sm_miscellaneous_information.pdf

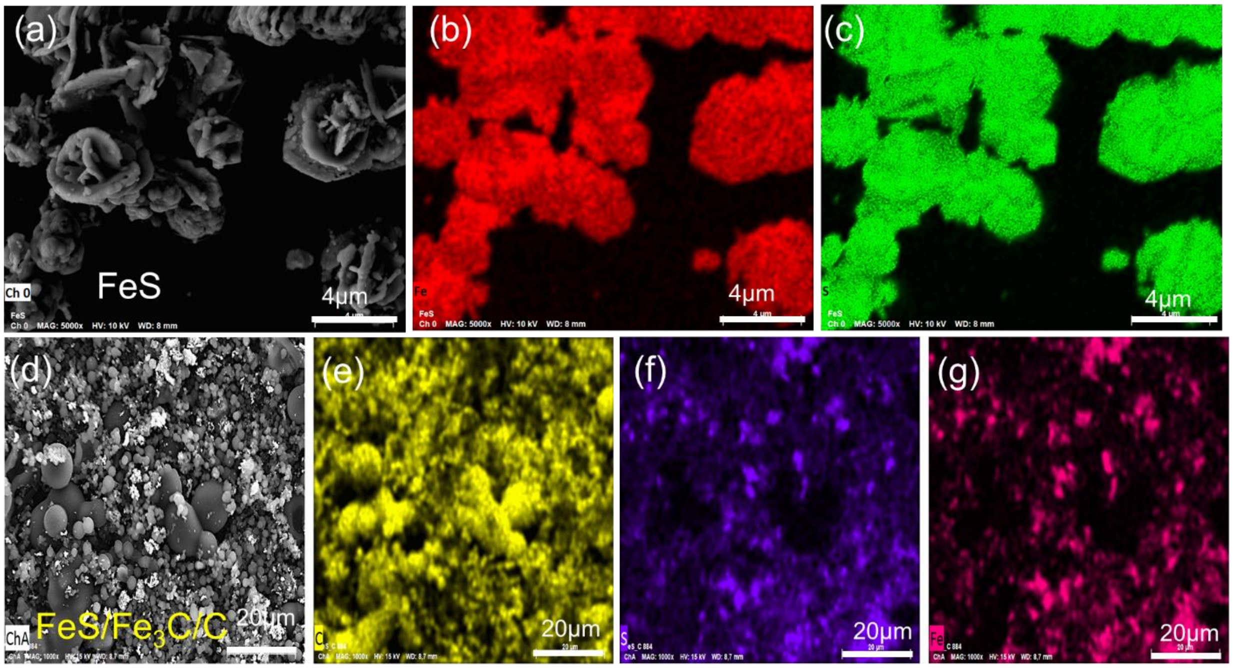

Figure. S1 The EDS elemental maps of the pristine FeS material (a, b, and c); correspondingly, the EDS of the pristine FeS/Fe_3C/C material (d, e, f, and g).

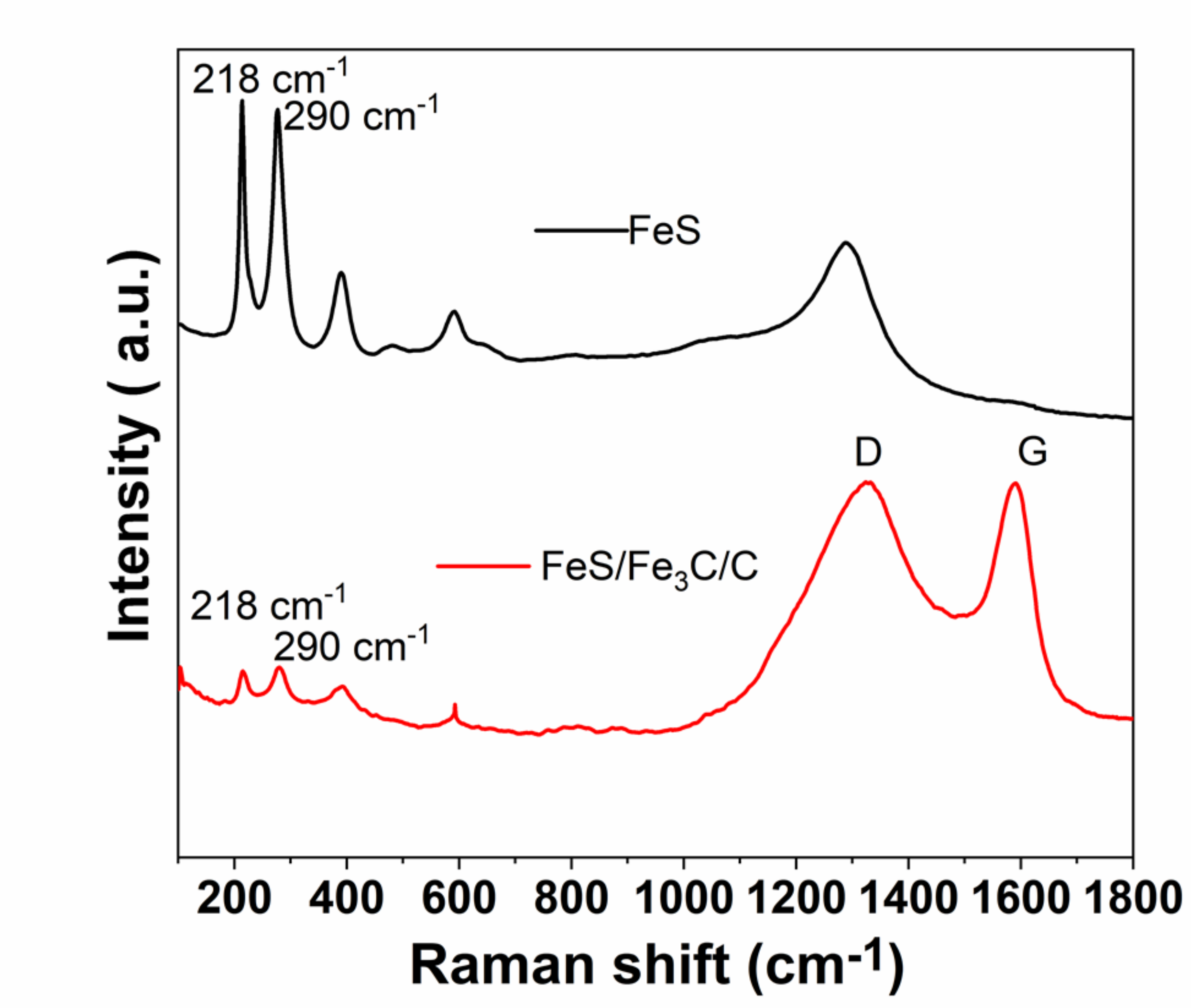

Figure. S2 The Raman spectra the pristine FeS material (black) and the pristine $FeS/Fe_3C/C$ material (red).

Table. 1 Organic Elemental Analysis (OEA) of the pristine FeS and $FeS/Fe_3C/C$ material

	N (%)	C (%)	H (%)	S (%)
FeS	0.07	0.32	0.610	40.84
$FeS/Fe_3C/C$	0.36	57.76	1.258	16.508

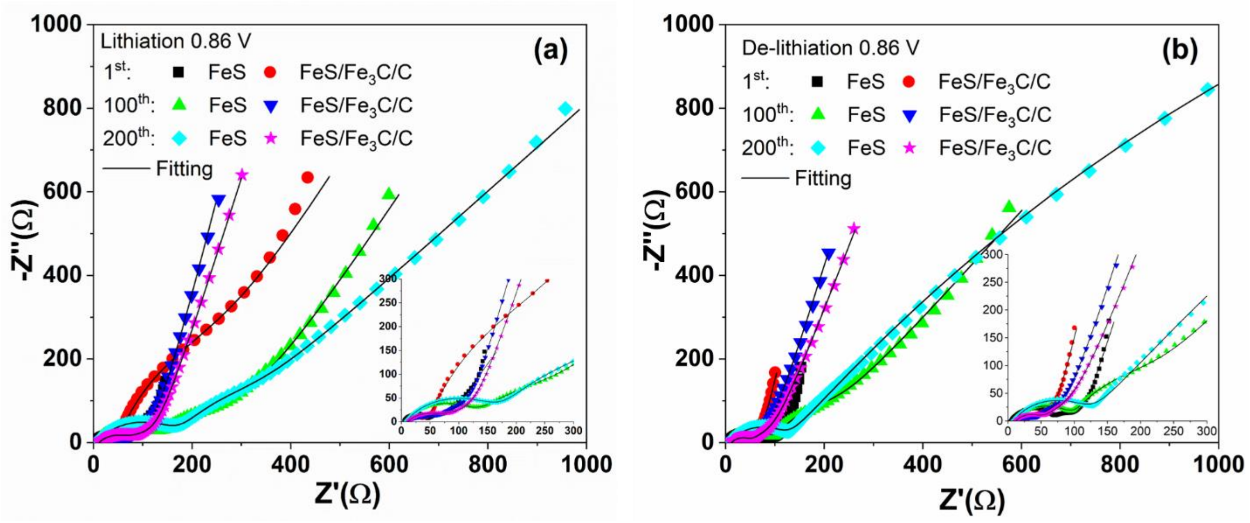

Figure. S3 The Nyquist plots of FeS and FeS/Fe_3C/C electrodes at some selected cycles (1st, 100th, and 200th) in lithiation (a) and de-lithiation conditions (b).
